# Analyzing the Estrogen Receptor Status of Liver Metastases with [^18^F]-FES-PET in Patients with Breast Cancer

**DOI:** 10.3390/diagnostics11112019

**Published:** 2021-10-30

**Authors:** Jorianne Boers, Naila Loudini, Robbert J. de Haas, Antoon T. M. Willemsen, Bert van der Vegt, Elisabeth G. E. de Vries, Geke A. P. Hospers, Carolina P. Schröder, Andor W. J. M. Glaudemans, Erik F. J. de Vries

**Affiliations:** 1 Department of Medical Oncology, University Medical Center Groningen, University of Groningen, 9713 Groningen, The Netherlands; j.boers@umcg.nl (J.B.); e.g.e.de.vries@umcg.nl (E.G.E.d.V.); g.a.p.hospers@umcg.nl (G.A.P.H.); c.p.schroder@umcg.nl (C.P.S.); 2 Department of Nuclear Medicine and Molecular Imaging, University Medical Center Groningen, University of Groningen, 9713 Groningen, The Netherlands; n.loudini@student.rug.nl (N.L.); a.t.m.willemsen@umcg.nl (A.T.M.W.); a.w.j.m.glaudemans@umcg.nl (A.W.J.M.G.); 3 Department of Radiology, University Medical Center Groningen, University of Groningen, 9713 Groningen, The Netherlands; r.j.de.haas@umcg.nl; 4 Department of Pathology, University Medical Center Groningen, University of Groningen, 9713 Groningen, The Netherlands; b.van.der.vegt@umcg.nl

**Keywords:** FES-PET/CT, breast cancer, liver metastases, estrogen receptor, quantification

## Abstract

Background: Positron emission tomography (PET) with 16α-[^18^F]-fluoro-17β-estradiol ([^18^F]-FES) can visualize estrogen receptor (ER) expression, but it is challenging to determine the ER status of liver metastases, due to high physiological [^18^F]-FES uptake. We evaluated whether [^18^F]-FES-PET can be used to determine the ER status of liver metastases, using corresponding liver biopsies as the gold standard. Methods: Patients with metastatic breast cancer (*n* = 23) were included if they had undergone a [^18^F]-FES-PET, liver metastasis biopsy, CT-scan, and [^18^F]-FDG-PET. [^18^F]-FES-PET scans were assessed by visual and quantitative analysis, tracer uptake was correlated with ER expression measured by immunohistochemical staining and the effects of region-of-interest size and background correction were determined. Results: Visual analysis allowed ER assessment of liver metastases with 100% specificity and 18% sensitivity. Quantitative analysis improved the sensitivity. Reduction of the region-of-interest size did not further improve the results, but background correction improved ER assessment, resulting in 83% specificity and 77% sensitivity. Using separate thresholds for ER+ and ER− metastases, positive and negative predictive values of 100% and 75%, respectively, could be obtained, although 30% of metastases remained inconclusive. Conclusion: In the majority of liver metastases, ER status can be determined with [^18^F]-FES-PET if background correction and separate thresholds are applied.

## 1. Introduction

Patients with breast cancer may develop distant metastases in the course of the disease, with the liver being a common metastatic site [1,2,3]. Most breast cancer tumors express the estrogen receptor (ER), which is an important guide for the selection of treatment. However, ER expression of metastases can change over time [4,5], and discordance in ER expression between the primary tumor and liver metastases has been observed in 14% of the patients [2]. In the case of metastatic disease, repeated histological biopsies are therefore recommended to re-evaluate the ER status immunohistochemically, which is the current gold standard [6]. However, assessment of ER status from tumor biopsies has important limitations, such as heterogeneity in ER expression within and between metastases, the invasive nature of the procedure with potentially significant complications (e.g., bleeding risk), technically challenges for lesions in complex locations, and sampling errors [7]. A non-invasive whole-body imaging method, such as 16α-[^18^F]-fluoro-17β-estradiol ([^18^F]-FES) positron emission tomography (PET), could potentially overcome these limitations. [^18^F]-FES-PET uses the in vivo binding of the radiolabeled estradiol analog to the ER to determine the ER status of all tumor lesions within a patient and thus can provide information about heterogeneity in ER expression [8].

However, [^18^F]-FES-PET also has some limitations, in particular related to the evaluation of liver metastases [9,10]. A recommendation paper on the correct use of [^18^F]-FES-PET concluded that this imaging technique is not an optimal tool to evaluate liver metastases, due to high physiological uptake as a result of rapid metabolism of the radiolabeled estrogen analog in the liver [9]. This recommendation paper suggested that low tumor [^18^F]-FES uptake, defined as lower than physiological background uptake in liver parenchyma, could indicate ER− metastases or benign cysts, whereas high tumor [^18^F]-FES uptake, defined as exceeding physiological background uptake in liver parenchyma, could indicate ER+ metastases. However, [^18^F]-FES uptake lower than liver parenchyma can also be observed in ER+ liver metastases [9]. In fact, a previous study qualitatively investigated [^18^F]-FES uptake in liver metastases in a limited number of patients and found that only a few ER+ liver metastases were detected by [^18^F]-FES-PET, indicating that the sensitivity of [^18^F]-FES-PET for detecting liver metastases was poor [10]. However, a major limitation of that study was the lack of corresponding liver biopsies to confirm the imaging results immunohistochemically. To date, the feasibility of assessing the ER status in liver metastases with [^18^F]-FES-PET has not been reported in other studies. Therefore, liver metastases are usually excluded from the analysis of [^18^F]-FES-PET studies [11].

The aim of this exploratory study was to evaluate whether the ER status of liver metastases in patients with breast cancer can be assessed with [^18^F]-FES-PET, using the ER expression measured immunohistochemically in corresponding tumor liver biopsies as the gold standard. We investigated whether quantitative analysis is a better method for determining ER status in liver metastases than visual analysis. We also investigated which outcome parameter for tracer uptake could best be used to differentiate ER+ and ER− status and what the effects of the region-of-interest (ROI) size and background correction are.

## 2. Materials and Methods

### 2.1. Study Design and Patients

Eligible patients were selected from the multicenter IMPACT breast trial (NCT01957332; *n* = 120 patients) at location University Medical Center Groningen (UMCG), or the single-center palbociclib/[^18^F]-FES-PET trial (NCT02806050; *n* = 31 patients). The detailed methods for the IMPACT breast trial, as well as the palbociclib/[^18^F]-FES-PET study have been published previously [12,13,14,15,16]. Both protocols were approved by the institutional review board and written informed consent was obtained from each patient. Patients from both studies were included in this retrospective analysis if they were diagnosed with non-rapidly progressive metastatic breast cancer, regardless of ER status, and received a [^18^F]-FES-PET scan, a contrast-enhanced computed tomography (CT) scan of the abdomen, an [^18^F]-fluorodeoxyglucose-PET ([^18^F]-FDG-PET) scan, and a histological biopsy of a liver metastasis at baseline. CT and [^18^F]-FDG-PET scans were used to determine the exact location of the biopsied liver metastasis on the [^18^F]-FES-PET scan. One patient with a liver biopsy without tumor cells at histopathology was excluded.

### 2.2. [^18^F]-FES-PET Imaging

ER antagonists were discontinued for at least five weeks before [^18^F]-FES-PET imaging. Patients did not have to fast before the scan. Whole-body [^18^F]-FES-PET acquisition started 60 min after the intravenous injection of ~200 MBq [^18^F]-FES. All [^18^F]-FES-PET scans were performed with an emission acquisition time of 3 min per bed position, using a Siemens Biograph mCT40 or mCT64 scanner (Siemens Healthineers, Knoxville, TN, USA). Low dose CT was acquired for attenuation and scatter correction. Reconstructions of the scans and quantification of tracer uptake were performed according to the European Association of Nuclear Medicine Research Limited (EARL) criteria [17]. PET scans used for visual analysis were reconstructed with a 2-mm spatial filter.

### 2.3. Image Analysis

[^18^F]-FES uptake was only analyzed in the liver metastasis, from which a biopsy was taken for ER assessment. PET images and biopsy location were retrospectively reviewed. An experienced abdominal radiologist (RdH) verified the exact location of the biopsied lesion, based on available contrast-enhanced CT and/or abdominal ultrasound images. Nuclear medicine physicians visually interpreted the [^18^F]-FES-PET scans. Their visual assessment of the ER status of the biopsied liver lesion was based on the tracer uptake in the tumor relative to the uptake in liver parenchyma and classified into three categories: (i) tumor uptake lower than that of background liver tissue, (ii) tumor uptake similar to background activity (i.e., no delineation of the lesion visible), and (iii) tumor uptake exceeding background activity. For quantitative analysis, a two-dimensional elliptical or circular ROI, which matched the CT-based largest and smallest diameter of the tumor as closely as possible, was drawn around each biopsied lesion. ROIs were drawn in [^18^F]-FES-PET images by two trained observers (NL and JB) using Syngo.via VB30 imaging software (Siemens Healthineers, Knoxville, TN, USA), and checked by an experienced nuclear medicine physician (AG). Quantification of tracer uptake was performed in an observer-blinded fashion for histopathology reports. A quantitative assessment of the tracer uptake in the tumor was performed, using the maximum (SUV_max_), average (SUV_mean_), and minimum (SUV_min_) standardized uptake value within the ROI as outcome parameters. To investigate the influence of the ROI size on tracer uptake, smaller ROIs with one-third (SUV_1/3_) and two-thirds (SUV_2/3_) of the original ROI diameters were drawn. Physiological background tracer uptake was determined by measuring the average SUV_mean_ of two ROIs with a diameter of 1.5–2.5 cm drawn in two healthy liver segments. Background correction was performed by calculating the tumor-to-background ratios (TBR), defined as the ratio between the SUV_max_, SUV_mean_, or SUV_min_ of a liver metastasis and the average SUV_mean_ of healthy liver tissue and presented as TBR_max_, TBR_mean_, and TBR_min_, respectively.

### 2.4. Ultrasound-Guided Biopsy and Histopathological Examination

According to standard clinical care procedures, all ultrasound-guided percutaneous 18G core needle liver biopsies were performed by experienced abdominal radiologists. ER status of the liver metastasis was determined by staining the formalin-fixed paraffin-embedded liver biopsies using the CONFIRM anti-ER (SP1, Roche) on an automated Benchmark Ultra platform (Roche). Metastases were deemed ER+ if ≥10% of the tumor cells showed nuclear staining, according to Dutch guidelines. The immunohistochemical evaluation of the ER status was used as the reference standard.

### 2.5. Statistical Analysis

Descriptive statistics were used for visual and quantitative PET analyses. When the results were normally distributed, continuous variables are expressed as mean ± standard deviation (SD); otherwise, median and interquartile range (IQR) are reported. Categorical variables are expressed as numbers and percentages. The Mann–Whitney U test was used to compare quantitative [^18^F]-FES uptake between ER+ and ER− metastases, because normal distribution of the data could not be proven with the Shapiro–Wilk test, Levene’s test, and Q-Q plot. The area under the receiver operating characteristic (ROC) curve (AUC) and the 95% confidence interval (95% CI) were used to measure the discriminative power of [^18^F]-FES-PET to separate ER+ from ER− metastases, and to calculate the resulting sensitivity and specificity. We also defined two additional cut-off values per PET parameter, corresponding to >90% sensitivity and >90% specificity, respectively, to conclusively assign an ER status to the majority of metastases [18]. The positive and negative predictive values (PPV and NPV, respectively) and the percentage of inconclusive metastases were calculated when applying these two cut-off values. Statistical significance was defined by a *p*-value ≤ 0.05. Analyses were performed using IBM SPSS Statistics for Windows, version 23 (IBM Corp., Armonk, NY, USA).

## 3. Results

### 3.1. Patients

Twenty-three female patients (mean age of 59 ± 9 years) with a liver metastasis with corresponding immunohistochemical results were included. The majority of patients had newly diagnosed metastatic breast cancer (*n* = 18) without previous systemic treatment (Table 1). The biopsied liver metastases were classified based on immunohistochemical results into ER+ (*n* = 17) and ER− (*n* = 6). In 3 out of 23 patients (13%), the ER status of the liver metastasis differed from the ER status of the primary tumor (these patients had an ER+ primary tumor and ER− metastasis). The percentage of ER+ cells in ER+ lesions ranged from 80% to 100%, and the percentage in ER− lesions ranged from 0% (*n* = 5) to 5% (*n* = 1). The median longest radiological tumor diameter of the biopsied liver metastases was 29 mm [range: 13 to 76 mm], and the median smallest tumor diameter was 23 mm [range: 12 to 62 mm]. Twenty patients underwent a biopsy before [^18^F]-FES-PET [range: 0 to 27 days], and three patients after [^18^F]-FES-PET [range: 4 to 75 days; two out of these three patients after start of treatment].

### 3.2. Visual Assessment of [^18^F]-FES Uptake

**Figure 1:**
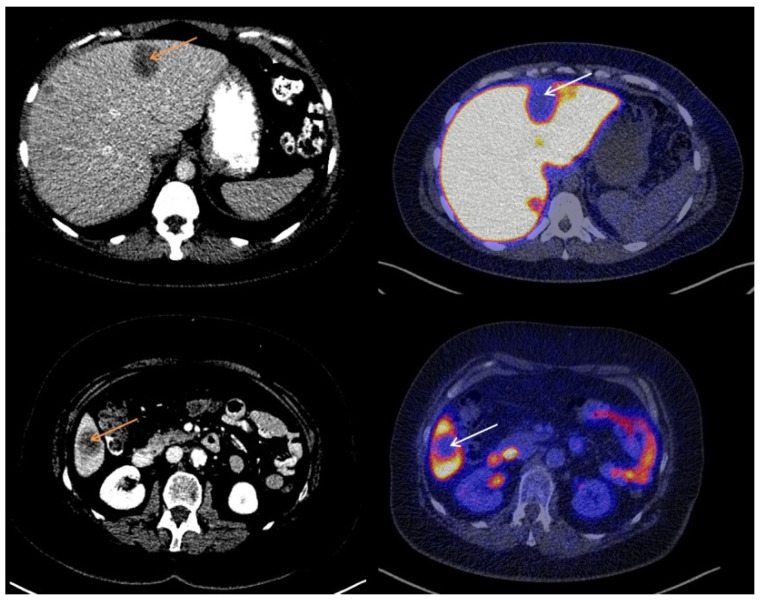
Two cases of low [^18^F]-FES uptake at the site of an ER− liver metastasis.

**Figure 2:**
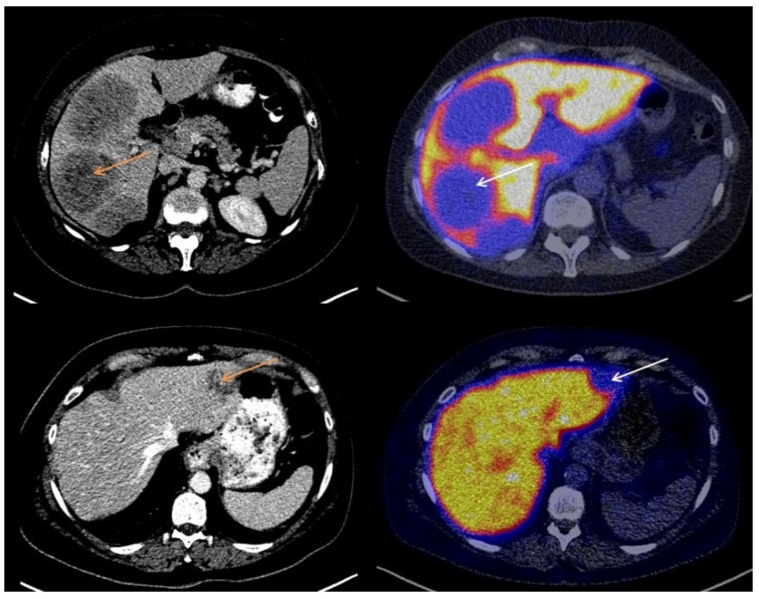
Two cases of low [^18^F]-FES uptake at the site of an ER+ liver metastasis.

**Figure 3:**
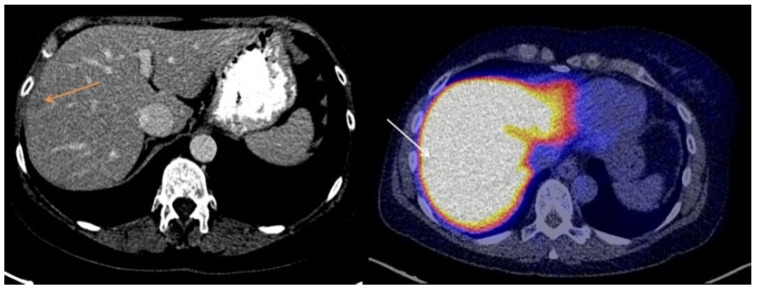
A case of an ER+ liver metastasis with [^18^F]-FES uptake equal to physiological liver uptake.

The majority of liver metastases detected with CT (20/23; 87%) showed visually lower tracer uptake on the [^18^F]-FES-PET scan than liver parenchyma. The other three liver metastases (13%) showed [^18^F]-FES uptake similar to background activity in the liver. No liver metastases with uptake higher than background liver activity were observed. Metastases with lower visual [^18^F]-FES uptake than healthy liver were classified as both ER+ (*n* = 14) and ER− metastases (*n* = 6). Metastases with tumor [^18^F]-FES uptake similar to background activity were all ER+ liver metastases (*n* = 3). Using immunohistochemical results as the gold standard, a sensitivity of 18% and a specificity of 100% were obtained for visual analysis of [^18^F]-FES-PET scans to assess ER expression in liver metastases. The negative predictive value was 30% (6 out of 20 metastases classified as having visually lower tumor [^18^F]-FES uptake than healthy liver were indeed ER−), and the positive predictive value was 100% (3 out of 3 metastases with tumor uptake similar to healthy liver were ER+). Figure 1 shows representative cases of low [^18^F]-FES uptake in an ER− liver metastasis. Figure 2 shows an example of an ER+ liver metastasis with lower uptake than liver. Figure 3 shows a case of an ER+ liver metastasis with [^18^F]-FES uptake similar to physiological liver uptake.

### 3.3. Quantitative Evaluation of [^18^F]-FES Uptake

**Figure 4:**
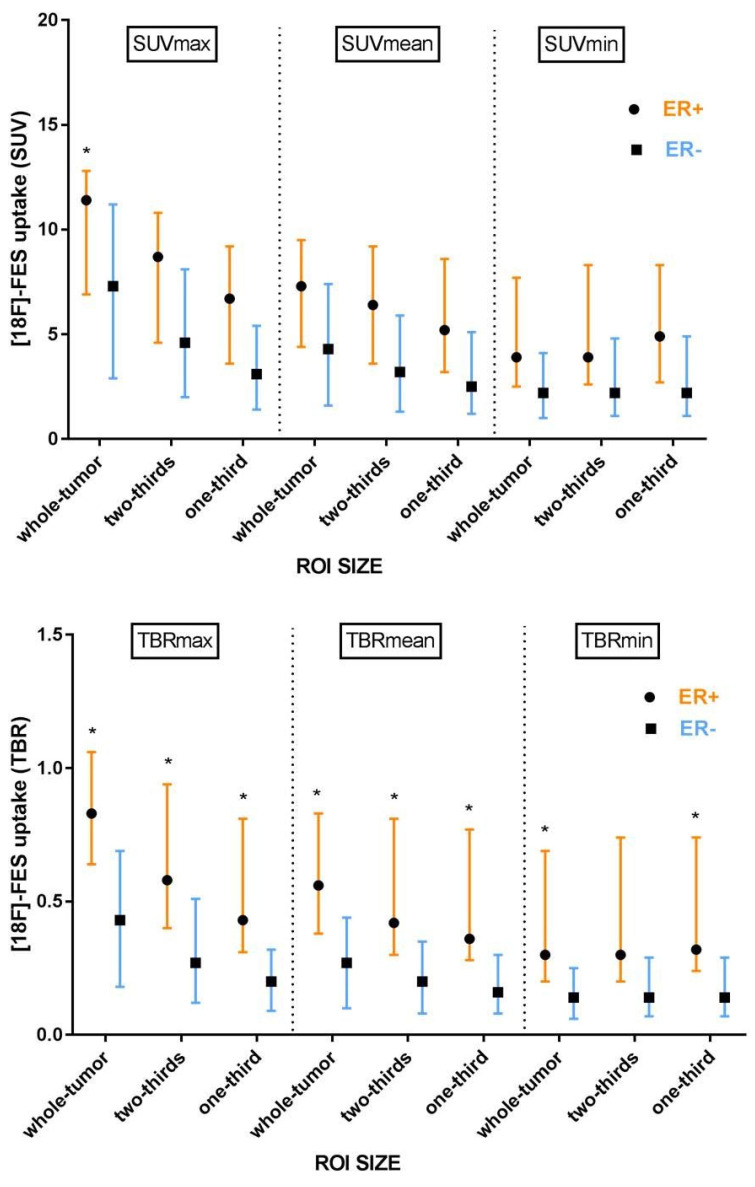
Tumor [^18^F]-FES uptake in ER+ and ER− liver metastases (*n* = 23; median and interquartile range), expressed as standardized uptake value (upper panel) or tumor-to-background ratio (lower panel). * p ≤ 0.05.

[^18^F]-FES uptake in liver metastases was first quantified, using an ROI that matched the CT-based size of the whole tumor. [^18^F]-FES uptake, expressed as SUV_max_, was significantly higher in ER+ liver metastases (median 11.4; IQR: 6.9–12.8) than in ER− liver metastases (median 7.3; IQR: 2.9–11.2; *p* = 0.050). [^18^F]-FES uptake in ER+ and ER− metastases was not significantly different anymore when tracer uptake in whole-tumor ROIs was expressed as SUV_mean_ or SUV_min_ (Figure 4; Table A1). ROC analysis for the tracer uptake in whole-tumor ROIs showed the highest AUC if [^18^F]-FES uptake was expressed as SUV_max_ (0.78 (95% CI: 0.55–1.0); Table 2). Quantitative assessment of [^18^F]-FES uptake using whole-tumor ROIs resulted in a higher sensitivity to detect ER+ liver metastases (SUV_max_, SUV_mean_, and SUV_min_: 53, 94, and 53%) than visual inspection (18%), although at the expense of a lower specificity for SUV_mean_ (50%), and SUV_min_ (83%), but not for SUV_max_ (100%) (Table 2).

### 3.4. The Effect of ROI Size

When using full-sized ROIs, partial-volume effects may affect the quantification of [^18^F]-FES uptake in liver metastases. Therefore, we investigated whether the impact of spill-in of the high background activity from liver parenchyma could be reduced by reducing the ROI size. Indeed, lower tumor [^18^F]-FES uptake, expressed as SUV_max_ and SUV_mean_, was seen for smaller ROIs (Figure 4; Table A1). However, [^18^F]-FES uptake in the metastasis, expressed as SUV_min_, was not affected by the reduction of ROI size, except for a slight increase in SUV_min_ in the smallest ROI of ER+ lesions. Decreasing the ROI size did not show a remarkable improvement in the results of the ROC analysis (AUC, Table 2). In general, a reduction in ROI size tended to improve the sensitivity to detect ER+ metastases for SUV_max_ and SUV_min_, but at the expense of specificity (Table 2). On the other hand, a reduction in ROI size tended to improve the specificity for SUV_mean_, but at the expense of sensitivity (Table 2).

### 3.5. Background Correction

Because of the potential spill-in effects on [^18^F]-FES uptake in liver metastases, the effect of background correction was explored. Surprisingly, differences in [^18^F]-FES uptake in the healthy liver were observed between ER subgroups. [^18^F]-FES uptake in healthy liver tissue in the ER− group was significantly higher compared to the ER+ group (SUV_mean_: 16.1 [15.6–16.5] vs. 13.0 [11.3–15.9], *p* = 0.030). Due to this difference in background tracer activity, a background correction may be required and therefore the TBR was calculated. Significantly higher TBRs in ER+ than in ER− liver metastases were observed for all measures, except for the TBR_min_ of the two-third sized ROI (Figure 4; Table A1). ROC analysis revealed that background correction resulted in a higher AUC for TBR_max_, TBR_mean,_ and TBR_min_ (Table 2), when compared to the SUV measures. Overall, the highest AUC was found for the TBR_max_ from a whole-tumor ROI. The optimal TBR_max_ cut-off value of ≥0.69, resulted in a sensitivity of 77% and specificity of 83% to detect ER+ liver metastases.

### 3.6. Lower and Upper Cut-Off Values

To improve the predictive value for the ER status of the majority of liver metastases, two cut-off values were defined, dividing the tumors into 3 groups: (1) lesions with tracer uptake exceeding or similar to the upper threshold, corresponding to ER+ liver metastases; (2) lesions with tracer uptake below the lower limit, corresponding to ER− liver metastases; and (3) lesions with tracer uptake between the upper and lower threshold, corresponding to equivocal liver metastases. The upper and lower threshold for all outcome parameters (SUV and TBR) were calculated to achieve a specificity and sensitivity >90%, respectively (Table 3). The highest positive and negative predictive value and lowest percentage of equivocal liver metastases were obtained if TBR_max_ from a whole-tumor ROI was used. When the optimal cut-off value of <0.33 was used for ER− metastases and ≥0.73 for ER+ metastases, the negative predictive value was 75% (three out of four metastases with a value <0.33 were ER−), and the positive predictive value was 100% (12 out of 12 metastases with a value ≥0.73 were ER+) (Table 3). Thirty percent of metastases had a TBR_max_ in-between these thresholds and therefore were defined as equivocal metastases (*n* = 7). If higher sensitivity and specificity would be required, the cut-off values could be adjusted accordingly, although at the expense of a higher percentage of equivocal lesions. At a sensitivity and specificity of 95%, for example, the percentage of equivocal metastases would increase to 35%.

## 4. Discussion

In this exploratory study, we found that the ER status of the majority of liver metastases in patients with breast cancer can be determined with [^18^F]-FES-PET, if quantitative analysis and correction for the background are applied.

**Figure 5:**
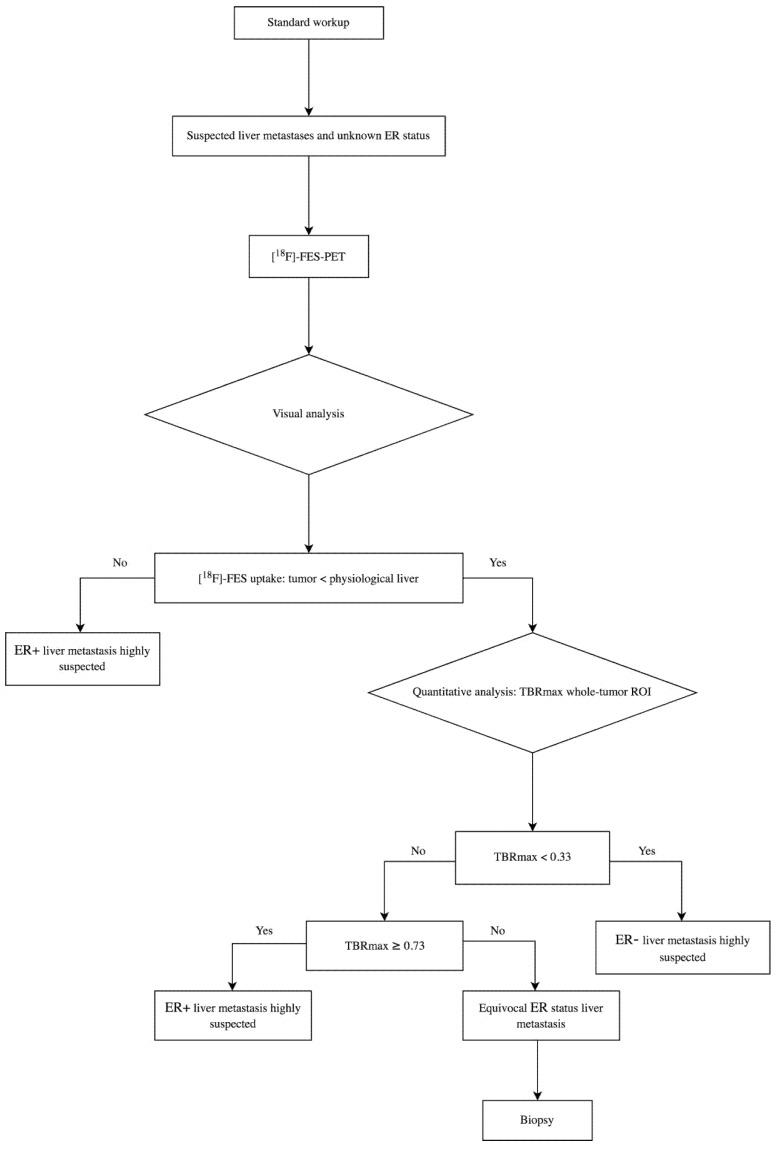
Decision flowchart: [^18^F]-FES-PET to define ER expression in liver metastases. Abbreviations: ER = estrogen receptor; TBR = tumor-to-background ratio; ROI = region-of-interest.

This is the first study comparing [^18^F]-FES uptake in a particular liver metastasis with immunohistochemistry in the matching liver biopsy for assessing the ER status. This is essential for future research settings since it would be beneficial if liver metastases can potentially be included in the analysis of [^18^F]-FES-PET studies. In daily practice, these data can help clinicians provide information on ER status of all metastases within a patient, including liver metastases. This is even more interesting in light of the recent FDA approval of [^18^F]-FES. Based on the results of this study, we developed a flowchart for determining the ER status of liver metastases with [^18^F]-FES-PET (Figure 5). Visual analysis is an important first step in detecting ER+ metastases. If the lesion shows [^18^F]-FES uptake at least equal to physiological liver uptake an ER+ metastasis is likely. However, as a second step, quantitative analysis is needed for metastases with [^18^F]-FES uptake lower than physiological liver uptake. Note that due to the high background signal in the liver, different thresholds are used for the characterization of liver metastases for non-liver lesions. The TBR_max_ is used for the final determination of ER+, ER− and equivocal liver metastases.

In 87% of the patients, the liver metastasis was visible on [^18^F]-FES-PET as a lesion with lower tracer uptake than the surrounding liver. In the other cases, the lesion was not visible due to similar [^18^F]-FES uptake as background liver activity. Our visual analysis is in line with other [^18^F]-FES-PET studies [10,19], and studies using other tracers [20]. Low tracer uptake in a liver lesion appears due to the high background uptake rather than the absence of ER expression.

Recently, a meta-analysis comparing [^18^F]-FES uptake with ER expression measured immunohistochemically in a biopsy sample, found a 78% overall sensitivity and 98% specificity to detect ER expression in non-liver metastases in patients with breast cancer. This meta-analysis included studies using visual assessment or quantitative analysis [21]. We found a similar sensitivity of 77%, but a slightly lower specificity of 83% to distinguish ER+ from ER− liver metastases, using the TBR_max_ from a whole-tumor ROI. In our analysis, quantitative approaches achieved more balanced sensitivity and specificity measures than a visual approach. Obviously, our results will have to be confirmed in a prospective study. Until such confirmation, the suggested cut-off values should be used with caution and cannot replace the gold standard for immunohistochemical analysis. The present study clearly shows problems with the use of [^18^F]-FES-PET to assess ER status of liver metastases. We have demonstrated an approach to overcome the shortcomings. Although it is not clear yet whether our methodology would be clinically useful, our results at least provide guidance for the classification of a part of the liver metastases with [^18^F]-FES-PET.

In this study, liver metastases were considered ER+ if at least 10% of the tumor cells in the biopsy sample showed nuclear staining, according to Dutch guidelines. Other guidelines (e.g., ASCO/CAP) use 1% of positively stained cells as a cut-off. In our study, only one subject had between 1% and 10% of positively stained cells. When 1-10% staining was considered as low-positive and a cut-off of ≥1% was used for ER positivity, only one subject would change from ER− to ER+. Yet, this change increased the AUC for TBR_max_ (whole-tumor ROI) from 0.87 to 0.91, the specificity from 83 to 100%, and the sensitivity from 77 to 78%, when an optimal single cut-off of 0.69 is used. When two thresholds are used (optimal cut-off: 0.33 and 0.69), the NPV and PPV are unaffected by the change in classification of the subject with a weak ER+ lesion, but a reduction in the number of equivocal lesions from 30 to 22% is observed (Table A2 and Table A3). The present study takes into account possible methodological issues, such as spillover effects. One potential solution to this problem is to reduce the ROI size. We found that SUV_max_ and SUV_mean_ increased with increasing ROI size. This can be explained by increasing spill-in of the higher signal from liver parenchyma with increasing ROI size [22]. At the border of the ROI, the liver metastasis contains a higher signal (including the maximum pixel) than the center of the lesion, because of the high physiological [^18^F]-FES uptake in the surrounding liver. The ROI size did not affect SUV_min_, with the exception of the SUV_min_ in the smallest ROI of ER+ lesions. This could be explained by the fact that the pixel with minimum uptake may not always be situated at the center of the lesion due to heterogeneous receptor expression within ER+ tumors, statistical noise, or the lesion being located at the subcapsular region. Overall, reduction of the ROI size did not substantially improve the performance of [^18^F]-FES-PET to assess ER status in liver metastases. Since small metastases are more susceptible to partial-volume effects than large metastases, we explored the effect of excluding metastases with a diameter <2 cm (*n* = 3), but this did not improve the results (AUC, sensitivity, specificity; Table A4). On the other hand, background correction did improve the discriminative power of [^18^F]-FES-PET, with the best quantitative measure for discriminating ER+ from ER− liver metastases being the TBR_max_ for whole-tumor ROIs. Therefore, we suggest using the TBR_max_ to determine ER status in liver metastases, rather than the SUV_max_ with a cut-off value of 1.5–2.0, which is the most commonly reported value in the literature for non-liver breast cancer metastases.

Remarkably, a difference in tracer uptake in healthy liver tissue between ER+ and ER− metastases was found. The [^18^F]-FES uptake in healthy liver tissue of patients with ER+ tumors in our study was similar to other studies in patients with (suspected) ER+ breast cancer (median SUV_max_ 15.4, range: 12.5–18.7; median SUV_mean_ 12, IQR: 10–14) [19,23,24]. However, no value of [^18^F]-FES uptake specifically in healthy liver parenchyma of patients with ER− tumors was reported in these published studies. The [^18^F]-FES uptake in healthy liver tissue was significantly lower in patients with ER+ metastases than patients with ER− metastases in the present study. One could speculate that if a large fraction of the tracer is bound to the receptor in ER+ metastases, the amount of tracer available for liver uptake and metabolism would be reduced. However, our findings do not support this theory, as we found no correlation between the total [^18^F]-FES uptake (defined as the sum of SUV_max_ of all evaluable non-liver metastases), and physiological [^18^F]-FES uptake in the liver (SUV_mean_) (r = −0.404; *p* = 0.062), although this may be due to the sample size (*n* = 22, one patient had only liver metastases). Furthermore, the amount of circulating sex hormone-binding globulin (SHBG) levels may also influence physiological liver [^18^F]-FES uptake. In the literature, a higher SHBG level has been associated with lower tumor [^18^F]-FES uptake [25,26,27]. Another study reported that [^18^F]-FES binding to SHBG will protect the tracer from metabolism [28]. In the present study, SHBG serum concentrations were available for seven patients (four ER+ and three ER− metastases), but SHBG levels did not differ between patients with ER+ liver metastases and ER− liver metastases (ER+: 62 ± 35 nmol/L vs. ER−: 67 ± 31 nmol/L). Although this could be due to the small sample size, previously reported data also show that SHBG levels are not related to ER status [29].

A limitation of the present study is the inclusion of both pretreated and untreated patients with metastatic breast cancer, as prior treatment could theoretically have affected [^18^F]-FES uptake. Another limitation is the relatively small sample size, although the number of subjects with liver biopsies and [^18^F]-FES-PET in this study is larger than in any other study. The present study’s main strength is the use of liver metastases with a corresponding biopsy and immunohistochemical results. Other strengths are the use of various ROI sizes, the inclusion of both immunohistochemically confirmed ER+ and ER− liver metastases, and all PET scans being performed with EARL/EANM-accredited camera systems.

## 5. Conclusions

In conclusion, this exploratory study showed that the ER status in most liver metastases can be determined with [^18^F]-FES-PET, if correction for background and separate thresholds for ER+ and ER− metastases are applied. Visual analysis of the PET images is useful if tracer uptake in a liver metastasis is at least equal to physiological [^18^F]-FES uptake in the liver, indicating that the metastasis expresses ER. Quantitative analysis may have additional value for metastases with [^18^F]-FES uptake lower than the healthy liver, enabling stratification of about 70% of metastases.

## Figures and Tables

**Table 1:** Baseline characteristics *.

	Total (*n* = 23)	ER+ (*n* = 17)	ER− (*n* = 6)
Age, years (mean ± SD)	59 ± 9	59 ± 9	60 ± 12
Previous palliative systemic treatment			
Yes	5 (22%)	5 (29%)	0
No	18 (78%)	12 (71%)	6 (100%)
Tumor diameter on CT, mm (median; min-max range)			
Longest	29 [13–76]	28 [13–67]	32 [22–76]
Smallest	23 [12–62]	22 [12–62]	25 [15–42]

* In two patients, a liver biopsy was performed after starting endocrine treatment (after three weeks, and after eight weeks). There were no statistically significant differences in age and tumor size between groups. Abbreviations: SD = standard deviation; ER = estrogen receptor; CT = computed tomography.

**Table 2:** The discriminative power of [^18^F]-FES-PET to stratify liver metastases by ER status (*n* = 23).

Parameter	ROI Size	AUC (95% CI)	Optimal Cut-Off ER+	Sensitivity (%)	Specificity (%)
SUV_max_	whole-tumor	0.78 (0.55–1.00)	≥11.4	53	100
SUV_mean_	whole-tumor	0.74 (0.51–0.97)	≥2.2	94	50
SUV_min_	whole-tumor	0.71 (0.48–0.93)	≥3.9	53	83
SUV_max_	two-thirds	0.76 (0.54–0.97)	≥7.8	65	83
SUV_mean_	two-thirds	0.74 (0.51–0.97)	≥5.7	59	83
SUV_min_	two-thirds	0.72 (0.48–0.95)	≥1.9	88	50
SUV_max_	one-third	0.76 (0.55–0.97)	≥5.4	59	83
SUV_mean_	one-third	0.77 (0.55–0.98)	≥5.0	59	83
SUV_min_	one-third	0.75 (0.53–0.96)	≥3.3	65	67
TBR_max_	whole-tumor	0.87 (0.72–1.00)	≥0.69	77	83
TBR_mean_	whole-tumor	0.80 (0.62–0.99)	≥0.45	65	100
TBR_min_	whole-tumor	0.78 (0.58–0.97)	≥0.21	77	67
TBR_max_	two-thirds	0.79 (0.60–0.99)	≥0.52	65	83
TBR_mean_	two-thirds	0.82 (0.64–1.00)	≥0.33	77	83
TBR_min_	two-thirds	0.76 (0.54–0.97)	≥0.21	77	67
TBR_max_	one-third	0.83 (0.67–1.00)	≥0.32	77	83
TBR_mean_	one-third	0.84 (0.68–1.00)	≥0.30	77	83
TBR_min_	one-third	0.80 (0.62–0.99)	≥0.21	82	67

Abbreviations: ER = estrogen receptor; ROI = region-of-interest; AUC = area under the ROC curve; CI = confidence interval; SUV = standardized uptake value; TBR = tumor-to-background ratio.

**Table 3:** PET parameters and cut-off values to determine ER status by [^18^F]-FES-PET (*n* = 23).

Parameter	ROI Size	Cut-Off ER− Sensitivity >90%	Cut-Off ER+ Specificity >90%	NPV *	PPV ^‖^	Equivocal Metastases (%) ^‡^
SUV_max_	whole-tumor	<4.9	≥11.4	75	90	39
SUV_mean_	whole-tumor	<2.2	≥7.7	75	100	48
SUV_min_	whole-tumor	<1.2	≥4.8	67	89	48
SUV_max_	two-thirds	<2.5	≥9.5	75	100	52
SUV_mean_	two-thirds	<1.7	≥6.9	75	100	57
SUV_min_	two-thirds	<1.3	≥5.2	67	100	57
SUV_max_	one-third	<1.6	≥6.5	67	100	48
SUV_mean_	one-third	<1.5	≥5.8	67	100	52
SUV_min_	one-third	<1.3	≥5.3	67	100	52
TBR_max_	whole-tumor	<0.33	≥0.73	75	100	30
TBR_mean_	whole-tumor	<0.14	≥0.45	75	92	30
TBR_min_	whole-tumor	<0.07	≥0.29	50	100	52
TBR_max_	two-thirds	<0.15	≥0.65	75	100	48
TBR_mean_	two-thirds	<0.10	≥0.42	67	90	43
TBR_min_	two-thirds	<0.08	≥0.37	67	100	57
TBR_max_	one-third	<0.10	≥0.39	67	100	39
TBR_mean_	one-third	<0.09	≥0.34	50	91	43
TBR_min_	one-third	<0.08	≥0.32	67	100	48

* Negative predictive value, and the lowest cut-off value was used for ER− metastases; ^‖^ Positive predictive value, and the highest cut-off value was used for ER+ metastases; ^‡^ All measurements in-between the cut-off values can be defined as equivocal metastases. Abbreviations: ER = estrogen receptor; ROI = region-of-interest; NPV = negative predictive value; PPV = positive predictive value; SUV = standardized uptake value; TBR = tumor-to-background ratio.

## Data Availability

The dataset used for the current study is available from the corresponding author on reasonable request.

## Appendix A

**Table A1:** [^18^F]-FES uptake in liver metastases per ER status and per PET outcome parameter.

Parameter	ROI Size	ER+ (*n* = 17) (Median [IQR])	ER− (*n* = 6) (Median [IQR])	*p*-Value
SUV_max_	whole-tumor	11.4 [6.9–12.8]	7.3 [2.9–11.2]	0.050
SUV_mean_	whole-tumor	7.3 [4.4–9.5]	4.3 [1.6–7.4]	0.093
SUV_min_	whole-tumor	3.9 [2.5–7.7]	2.2 [1.0–4.1]	0.141
SUV_max_	two-thirds	8.7 [4.6–10.8]	4.6 [2.0–8.1]	0.069
SUV_mean_	two-thirds	6.4 [3.6–9.2]	3.2 [1.3–5.9]	0.086
SUV_min_	two-thirds	3.9 [2.6–8.3]	2.2 [1.1–4.8]	0.123
SUV_max_	one-third	6.7 [3.6–9.2]	3.1 [1.4–5.4]	0.069
SUV_mean_	one-third	5.2 [3.2–8.6]	2.5 [1.2–5.1]	0.059
SUV_min_	one-third	4.9 [2.7–8.3]	2.2 [1.1–4.9]	0.080
TBR_max_	whole-tumor	0.83 [0.64–1.06]	0.43 [0.18–0.69]	0.008
TBR_mean_	whole-tumor	0.56 [0.38–0.83]	0.27 [0.10–0.44]	0.030
TBR_min_	whole-tumor	0.30 [0.20–0.69]	0.14 [0.06–0.25]	0.050
TBR_max_	two-thirds	0.58 [0.40–0.94]	0.27 [0.12–0.51]	0.036
TBR_mean_	two-thirds	0.42 [0.30–0.81]	0.20 [0.08–0.35]	0.021
TBR_min_	two-thirds	0.30 [0.20–0.74]	0.14 [0.07–0.29]	0.069
TBR_max_	one-third	0.43 [0.31–0.81]	0.20 [0.09–0.32]	0.017
TBR_mean_	one-third	0.36 [0.28–0.77]	0.16 [0.08–0.30]	0.014
TBR_min_	one-third	0.32 [0.24–0.74]	0.14 [0.07–0.29]	0.030

Abbreviations: ER = estrogen receptor; ROI = region-of-interest; IQR = interquartile range; SUV = standardized uptake value; TBR = tumor-to-background ratio.

**Table A2:** The discriminative power of [^18^F]-FES-PET to stratify liver metastases by ER status (*n* = 23), using a cut-off value of ≥1% for ER positivity.

Parameter	ROI Size	AUC (95% CI)	Optimal Cut-Off ER+	Sensitivity (%)	Specificity (%)
SUV_max_	whole-tumor	0.83 (0.63–1.00)	≥4.0	100	60
SUV_mean_	whole-tumor	0.76 (0.50–1.00)	≥2.2	94	60
SUV_min_	whole-tumor	0.73 (0.50–0.97)	≥1.9	83	60
SUV_max_	two-thirds	0.78 (0.55–1.00)	≥7.3	67	80
SUV_mean_	two-thirds	0.76 (0.51–1.00)	≥1.7	94	60
SUV_min_	two-thirds	0.73 (0.47–0.99)	≥1.9	89	60
SUV_max_	one-third	0.78 (0.56–1.00)	≥2.4	89	60
SUV_mean_	one-third	0.78 (0.54–1.00)	≥1.5	94	60
SUV_min_	one-third	0.75 (0.53–0.96)	≥3.3	65	67
TBR_max_	whole-tumor	0.91 (0.78–1.00)	≥0.69	78	100
TBR_mean_	whole-tumor	0.82 (0.63–1.00)	≥0.45	61	100
TBR_min_	whole-tumor	0.77 (0.55–0.99)	≥0.13	83	60
TBR_max_	two-thirds	0.82 (0.61–1.00)	≥0.41	78	80
TBR_mean_	two-thirds	0.82 (0.62–1.00)	≥0.33	72	80
TBR_min_	two-thirds	0.74 (0.49–1.00)	≥0.12	89	60
TBR_max_	one-third	0.83 (0.66–1.00)	≥0.32	72	80
TBR_mean_	one-third	0.83 (0.65–1.00)	≥0.30	72	80
TBR_min_	one-third	0.79 (0.58–1.00)	≥0.13	89	60

Abbreviations: ER = estrogen receptor; ROI = region-of-interest; AUC = area under the ROC curve; CI = confidence interval; SUV = standardized uptake value; TBR = tumor-to-background ratio.

**Table A3:** PET parameters and cut-off values to determine ER status by [^18^F]-FES-PET (*n* = 23), using a cut-off value of ≥1% for ER positivity.

Parameter	ROI Size	Cut-Off ER− Sensitivity > 90%	Cut-Off ER+ Specificity > 90%	NPV *	PPV ^‖^	Equivocal Metastases (%) ^‡^
SUV_max_	whole-tumor	<4.9	≥11.3	75	100	39
SUV_mean_	whole-tumor	<2.2	≥7.7	75	100	48
SUV_min_	whole-tumor	<1.2	≥4.8	50	100	57
SUV_max_	two-thirds	<2.5	≥9.5	75	100	52
SUV_mean_	two-thirds	<1.7	≥6.9	75	100	57
SUV_min_	two-thirds	<1.3	≥5.2	67	100	57
SUV_max_	one-third	<1.6	≥6.5	67	100	48
SUV_mean_	one-third	<1.5	≥5.8	67	100	61
SUV_min_	one-third	<1.3	≥5.3	67	100	52
TBR_max_	whole-tumor	<0.33	≥0.69	75	100	22
TBR_mean_	whole-tumor	<0.11	≥0.45	50	92	39
TBR_min_	whole-tumor	<0.07	≥0.29	50	100	52
TBR_max_	two-thirds	<0.15	≥0.65	75	100	48
TBR_mean_	two-thirds	<0.10	≥0.42	75	100	44
TBR_min_	two-thirds	<0.08	≥0.37	67	100	57
TBR_max_	one-third	<0.10	≥0.39	50	100	35
TBR_mean_	one-third	<0.09	≥0.34	60	91	30
TBR_min_	one-third	<0.08	≥0.32	67	100	35

* Negative predictive value, and the lowest cut-off value was used for ER− metastases; ^‖^ Positive predictive value, and the highest cut-off value was used for ER+ metastases; ^‡^ All measurements in-between the cut-off values can be defined as equivocal metastases. Abbreviations: ER = estrogen receptor; ROI = region-of-interest; NPV = negative predictive value; PPV = positive predictive value; SUV = standardized uptake value; TBR = tumor-to-background ratio.

**Table A4:** The discriminative power of [^18^F]-FES-PET to stratify liver metastases by ER status (*n* = 20 metastases with a longest diameter ≥ 2 cm).

Parameter	ROI Size	AUC (95% CI)	Optimal Cut-Off ER+	Sensitivity (%)	Specificity (%)
SUV_max_	whole-tumor	0.74 (0.49–0.99)	≥4.0	100	50
SUV_mean_	whole-tumor	0.68 (0.41–0.95)	≥2.2	93	50
SUV_min_	whole-tumor	0.63 (0.36–0.90)	≥1.9	79	50
SUV_max_	two-thirds	0.71 (0.47–0.96)	≥7.8	57	83
SUV_mean_	two-thirds	0.69 (0.42–0.96)	≥1.7	93	50
SUV_min_	two-thirds	0.66 (0.38– 0.92)	≥1.9	86	50
SUV_max_	one-third	0.70 (0.46–0.95)	≥2.4	86	50
SUV_mean_	one-third	0.71 (0.47–0.96)	≥1.5	93	50
SUV_min_	one-third	0.69 (0.44–0.94)	≥1.9	86	50
TBR_max_	whole-tumor	0.85 (0.67–1.00)	≥0.69	71	83
TBR_mean_	whole-tumor	0.76 (0.54–0.98)	≥0.45	57	100
TBR_min_	whole-tumor	0.73 (0.50–0.96)	≥0.21	71	67
TBR_max_	two-thirds	0.75 (0.52–0.98)	≥0.41	71	67
TBR_mean_	two-thirds	0.79 (0.57–1.00)	≥0.33	71	83
TBR_min_	two-thirds	0.70 (0.45–0.96)	≥0.21	71	67
TBR_max_	one-third	0.80 (0.60– 0.99)	≥0.32	71	83
TBR_mean_	one-third	0.81 (0.61–1.00)	≥0.30	71	83
TBR_min_	one-third	0.76 (0.54–0.98)	≥0.21	79	67

Abbreviations: ER = estrogen receptor; ROI = region-of-interest; AUC = area under the ROC curve; CI = confidence interval; SUV = standardized uptake value; TBR = tumor-to-background ratio.
